# Saudi dental students’ perceptions of pediatric behavior guidance techniques

**DOI:** 10.1186/s12909-015-0382-6

**Published:** 2015-09-10

**Authors:** Asma M. Al-Jobair, Manal A. Al-Mutairi

**Affiliations:** Department of Pediatric Dentistry and Orthodontics, College of Dentistry, King Saud University, P.O. Box 60169, Riyadh, 11545 Saudi Arabia

**Keywords:** Saudi dental students, Behavior guidance techniques, Pediatric dentistry, Dental education

## Abstract

**Background:**

Dental students receive theoretical and clinical training in pediatric behavioral guidance techniques at university. Therefore, the content of the educational course and the degree of training in behavioral techniques may have an impact on the students’ perceptions and practice of such techniques. The purpose of this study was to evaluate Saudi dental students’ perceptions of behavior guidance techniques used in pediatric dentistry, and to assess the changes in their perceptions after 1 academic year of a didactic and clinical educational course.

**Methods:**

This longitudinal study was carried out once at the beginning and once at the end of the 2013/2014 academic year at the College of Dentistry, King Saud University in Riyadh, Saudi Arabia. A questionnaire measuring the perceived acceptability of behavior guidance techniques was completed by 78 fourth-year dental students before and after a pediatric dental course. Acceptability ratings were scored on a 5-point Likert scale and compared and evaluated in relation to demographic data. Paired *t*-test and one-way analysis of variance were used for the statistical analyses.

**Results:**

Before the course, the highest scores were for reinforcement and desensitizing techniques and the lowest were for aversive and communicative techniques. After the course, statistically significant increases were found in the acceptability of aversive techniques (voice control and hand-over-mouth), all pharmacological techniques, and modeling. Most communicative techniques and clinical situations were also rated as significantly more acceptable. Statistically significant decreases in acceptability ratings were found in promising a toy, and immobilization by staff or a parent. Immobilization using a papoose board, modeling, the presence of parents during the child’s treatment, and most communicative techniques were rated as significantly more acceptable by male students than female students.

**Conclusions:**

In general, Saudi dental students rated most basic behavior guidance techniques as acceptable. An educational course, including didactic and clinical components, improved their acceptability ratings, and had a considerable influence on their perceptions of behavior guidance in pediatric dentistry.

## Background

Working with uncooperative children is considered one of the most challenging experiences because the dentist’s clinical and management skills are truly tested. It is important that dentists have a wide range of behavior guidance techniques to meet the needs of the individual child, and are tolerant and flexible in the implementation of these techniques [1]. Children exhibit a broad range of physical, intellectual, emotional, and social attributes, accompanied by a diverse range of attitudes and temperaments [1]. Therefore, understanding children’s behavior and development is required to achieve success in managing and treating pediatric dental patients. The American Academy of Pediatric Dentistry (AAPD) has issued a set of guidelines on behavior guidance for the pediatric dental patient. Successful implementation of these guidelines enables the oral health team to perform quality treatment safely and efficiently, nurturing a positive dental attitude in the child [2]. Thus, the AAPD has recommended an increased focus on behavior guidance techniques during the entire period of dental education [3].

Dental students receive theoretical and clinical training in behavioral guidance techniques at university. Therefore, the content of the educational course and the degree of training in behavioral techniques may have an impact on students’ perceptions and practice of such techniques. According to Lewis [4], perceptions are dynamic, such that previously held and current perceptions may be changed because of the emergence of new and valid information. Several factors are responsible for differences in perception such as culture, background, education, and training [4]. In this context, the AAPD removed the hand-over-mouth technique from the list of recommended advanced behavioral guidance techniques in 2006 [5]. This change was made because of the controversial nature of the technique, the risks associated with its use, and decreased acceptability among parents and dental professionals.

The effect of education on dental students’ perceptions of behavior guidance techniques in pediatric dentistry was previously investigated by Sotto et al. [6]. They reported an increase in the acceptability of aversive behavior guidance, sedation, general anesthesia, and modeling among first-year dental students after an educational course. Bimstein et al. [7] also reported that at the end of the dental curriculum, students’ highest acceptability ratings were for positive reinforcement, use of nitrous oxide, stimulating the child’s imagination, and tell-show-do, whereas the lowest scores were for showing the needle to the child, and treatment without a local anesthetic. Additionally, when comparing attitudes after didactic and clinical training with those following didactic education alone, they reported a significant increase in the acceptability of general anesthesia and a significant decrease for situations involving the parent in the clinic [7]. Regarding behavior guidance components in different pre-doctoral pediatric dentistry programs, a survey found that almost all responding programs taught communicative techniques, protective stabilization, and pharmacologic techniques as being acceptable [8].

Several published studies have examined the attitude of Saudi parents towards behavior guidance techniques and the use of behavior guidance techniques by dentists in Saudi Arabia [9–11]. However, Saudi dental students’ perceptions of behavior guidance techniques have not yet been evaluated. Moreover, many studies have evaluated dental students’ acceptability ratings of behavior guidance techniques after having behavior guidance courses in different dental school curricula [6–8]. The acceptability of these techniques among dental students in the dental curriculum of King Saud University needs investigating.

The purpose of this study was to evaluate Saudi dental students’ perceptions of behavior guidance techniques used in pediatric dentistry, and to assess the changes in their perceptions after 1 academic year of didactic and clinical educational courses. Understanding and assessment of the students’ perceptions regarding these techniques provides an indication as to how and to what extent this educational material can be modified in dental programs.

## Methods

This longitudinal study was carried out at the beginning and the end of the 2013/2014 academic year at the College of Dentistry, King Saud University in Riyadh, Saudi Arabia, after the approval of the Ethics Committee of the College of Dentistry Research Center.

The undergraduate Bachelor of Dental Surgery program at King Saud University is 5 years long, followed by 1 year of an obligatory internship. In the current curriculum, dental students receive three pediatric dentistry courses over a 2.5-year period, starting in the second semester of the third year. The first course consists of didactic and practical components with no patient contact. The second course is taught in the fourth year and consists of didactic and clinical components. In this course, different lectures in non-pharmacological behavior guidance techniques are given in the first semester whereas pharmacological behavior guidance technique lectures are given in the second semester, and all lectures are delivered by different lecturers. In the first introductory clinic, a live demonstration is provided by the faculty on how to welcome the patients and their parents, how to seat the child, how to take the history and complete the paperwork, how to examine the child extra- and intra-orally, and how to administer local anesthesia. In the clinic, during 30 weekly sessions, all students have the opportunity to examine patients, provide preventive care, and perform various types of treatment on pediatric patients under the supervision of a specialized pediatric dentistry member of staff. The ratio of patients to students is one to one, while the average ratio of faculty to students is one to five. Treatments include, but are not limited to, restorations, pulp therapy, extraction, and space management. Students are allowed to treat healthy children aged 9–12 years who are relatively cooperative and communicative. They are exposed personally to basic behavior guidance techniques such as reinforcement, desensitization, and communicative techniques. Advanced behavior guidance techniques such as aversive and pharmacological techniques are not practiced clinically by undergraduate students. If the child shows a lack of cooperation while being treated by a fourth-year student, the supervising faculty member intervenes and tries the basic behavior guidance techniques as required in front of the student. If the child is still uncooperative, the patient is referred to a postgraduate pediatric dental student or to a pediatric dentist in the university.

A questionnaire used in a previous study by Sotto et al. [6] was used in the present study with some modifications to obtain students’ acceptability scores for pediatric dental behavior guidance techniques. The first part of the questionnaire required demographic data and information relating to gender, marital status, parental status, and previous dental and medical experiences. Demographic data were collected to assess the possible effects of these factors on the students’ perceptions. The second part of the questionnaire contained two sets of questions. The first set evaluated the acceptability of different behavior guidance techniques used in pediatric dentistry. Each technique was explained simply, in one line of text, and students were asked to assess the described technique on a scale ranging from “completely unacceptable” to “completely acceptable”. A second set of questions was used to assess the acceptability of clinical behavior scenarios from “never” to “always.”

Scores for 26 statements were marked on a 5-point Likert scale as follows: 1 = completely unacceptable/never; 2 = unacceptable/rarely; 3 = neutral/sometimes; 4 = acceptable/frequently; 5 = completely acceptable/always. The questionnaires were distributed to all fourth-year students (116 students) by the same investigator, once at the beginning of the first semester and once at the end of the second semester. Participation was voluntary and the responses were kept unidentified, although students were asked to write their university numbers so that the pre- and post- questionnaires could be matched.

Means and standard deviations of the scores were calculated before and after the course. Paired t-tests were used to evaluate the statistical significance of the change between pre- and post-course scores for each of the questionnaire items. A one-way analysis of variance was used to evaluate significant associations between the demographic data and the change in acceptability ratings. Statistical analyses were performed using Statistical Package for Social Sciences 20 software (SPSS Inc., Chicago, IL, USA). A significance level of 0.05 was adopted.

## Results

One hundred and one students returned the pre-course questionnaires out of 116 students. Three questionnaires were excluded because of incomplete information, giving a total of 98 questionnaires (84.5 %). After the course, 85 students returned the questionnaires. Seven questionnaires were excluded: four because the respondents had not completed the pre-course questionnaire, and three because they could not be matched with the pre-course questionnaires. Thus, only 78 pairs of the pre- and post-questionnaires (67.25 %) were suitable for analysis.

The demographic data of the respondents are presented in Table 1. The sample consisted of 35 female (45 %) and 43 male (55 %) students with an average age of 22.2 years. The majority of the respondents have older (71.8 %) and younger (93.6 %) siblings. Only three students (3.8 %) are married with children. Most of the students had received dental (92.3 %) and medical (74.4 %) treatment in the past themselves. Of these, the majority reported having had a pleasant dental (87.5 %) or medical (82.8 %) experience.

**Table 1:** Demographic data of the respondents, by number and percentage

Demographic Data		
Gender	Female	Male
	35 (45 %)	43 (55 %)
Age	22.2 Years	
Demographic Question	Yes (%)	No (%)
Do you have older siblings?	56 (71.8 %)	22 (28.2 %)
Do you have younger siblings?	73 (93.6 %)	5 (6.4 %)
Are you married?	3 (3.8 %)	75 (96.2 %)
Are you a parent?	3 (3.8 %)	75 (96.2 %)
Do you have a family member who is a dentist?	12 (15.4 %)	66 (84.6 %)
Have you received dental treatment?	72 (92.3 %)	6 (7.7 %)
Have you received medical treatment?	58 (74.4 %)	20 (25.6 %)
Was your dental experience pleasant?	63 (87.5 %)	9 (12.5 %)
Was your medical experience pleasant?	48 (82.8 %)	10 (17.2 %)

**Fig. 1:**
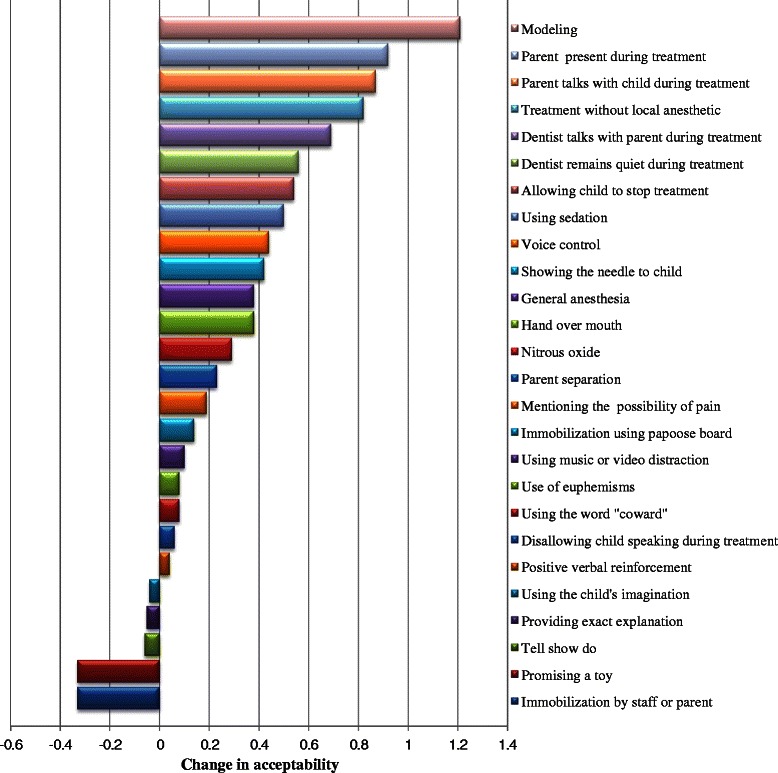
Graphical demonstration of the change in acceptability of behavior guidance techniques: positive values indicate increased acceptability

According to criteria outlined by Sotto et al. [6], behavior guidance techniques and clinical situations were categorized into six categories. The means and standard deviations of acceptability scores of different behavior guidance techniques and clinical situations before and after the course are shown in Table 2. All mean changes in the acceptability scores between pre- and post-course questionnaires are shown in Table 2 and graphically depicted in Fig. 1 in descending order.

**Table 2:** Means and standard deviations of pre- and post-course acceptability scores and the mean changes between them

Technique or situation	Pre-course scores	Post-course scores	Change		P value*
	Mean ± SD	Mean ± SD	Mean ± SD	(95 % CI)	
Reinforcement techniques					
Positive verbal reinforcement	4.13 ± 0.85	4.17 ± 0.79	0.04 ± 1.01	(−0.19 – 0.26)	NS
Promising a toy	4.06 ± 0.74	3.73 ± 0.94	−0.33 ± 1.11	(−0.58 – −0.08)	0.01
Using the word “coward”	3.53 ± 1.10	3.60 ± 1.04	0.07 ± 1.07	(−0.16 – 0.32)	NS
Overall score	3.91 ± 0.61	3.83 ± 0.61	– 0.07 ± 0.74	(−0.24 – 0.09)	NS
Aversive techniques					
Voice control	3.23 ± 1.18	3.67 ± 0.90	0.43 ± 1.35	(0.18 – 0.69)	0.001
Hand-over-mouth	1.96 ± 1.09	2.35 ± 1.18	0.38 ± 1.44	(0.06 – 0.70)	0.021
Immobilization by staff or parent	2.79 ± 0.88	2.46 ± 0.89	−0.33 ± 0.94	(−0.54 – −0.11)	0.003
Immobilization using papoose board	2.15 ± 0.82	2.29 ± 0.95	0.14 ± 1.05	(−0.09 – 0.37)	NS
Overall score	2.45 ± 0.67	2.69 ± 0.65	0.16 ± 0.66	(0.01 – 0.30)	0.04
Desensitization techniques					
Tell-show-do	4.06 ± 0.94	4.00 ± 1.05	−0.06 ± 0.15	(−0.36 – 0.23)	NS
Providing exact explanation	3.72 ± 0.85	3.67 ± 0.98	−0.05 ± 1.16	(−0.31 – 0.21)	NS
Using music or video distraction	4.01 ± 0.76	4.12 ± 0.93	0.10 ± 0.93	(−0.10 – 0.31)	NS
Using the child’s imagination	4.18 ± 0.83	4.14 ± 0.84	−0.04 ± 0.84	(−0.22 – 0.15)	NS
Use of euphemisms	3.90 ± 0.89	3.97 ± 0.92	0.07 ± 0.96	(−0.14 – 0.29)	NS
Modeling	2.71 ± 1.01	3.91 ± 0.95	1.20 ± 1.19	(0.93 – 1.47)	< 0.0001
Overall score	3.76 ± 0.46	3.97 ± 0.55	0.21 ± 0.56	(−0.01 – 0.38)	NS
Pharmacological techniques					
Nitrous oxide	3.54 ± 0.76	3.83 ± 0.82	0.29 ± 0.98	(0.07 – 0.51)	0.01
Using sedation	2.92 ± 0.97	3.42 ± 0.93	0.50 ± 1.17	(0.23 – 0.76)	< 0.0001
General anesthesia	2.73 ± 1.11	3.12 ± 1.18	0.38 ± 1.30	(0.09 – 0.67)	< 0.0001
Overall score	3.06 ± 0.66	3.46 ± 0.75	0.39 ± 0.74	(0.22 – 0.56)	< 0.0001
Communicative techniques					
Disallowing child speaking during treatment	2.36 ± 0.85	2.42 ± 0.93	0.06 ± 1.24	(−0.21 – 0.34)	NS
Mentioning the possibility of pain	3.24 ± 0.94	3.44 ± 0.81	0.19 ± 1.18	(−0.07 – 0.45)	NS
Treatment without local anesthetic	1.26 ± 0.54	2.08 ± 1.01	0.82 ± 1.13	(0.56 – 1.07)	< 0.0001
Allowing child to stop treatment	2.79 ± 0.95	3.33 ± 1.10	0.54 ± 1.28	(0.24 – 0.82)	< 0.0001
Dentist talks with parent during treatment	2.23 ± 0.93	2.92 ± 1.05	0.69 ± 1.22	(0.40 – 0.97)	< 0.0001
Dentist remains quiet during treatment	2.18 ± 0.89	2.74 ± 0.93	0.56 ± 1.27	(0.27 – 0.85)	< 0.0001
Parent talks with child during treatment	2.12 ± 0.73	2.99 ± 1.01	0.87 ± 1.17	(0.60 – 1.13)	< 0.0001
Overall score	2.31 ± 0.36	2.82 ± 0.45	0.53 ± 0.62	(0.39 – 0.67)	< 0.0001
Clinical situations					
Parent separation	2.94 ± 1.15	3.17 ± 1.15	0.23 ± 1.28	(−0.6 – 0.52)	NS
Parent present during treatment	2.68 ± 0.86	3.60 ± 0.93	0.92 ± 1.16	(0.66 – 1.18)	< 0.0001
Showing the needle to the child	1.10 ± 0.34	1.53 ± 0.92	0.42 ± 0.97	(0.20 – 0.64)	< 0.0001
Overall score	2.24 ± 0.47	2.76 ± 0.43	0.52 ± 0.57	(0.39 – 0.65)	< 0.0001

*SD* standard deviation, *CI* confidence interval of the difference

*Paired *t*-test, NS= not significant, P>0.05

Table 2 shows that before the course, the most acceptable approaches were reinforcement and desensitization techniques, with no significant change in their overall acceptability ratings after the course. However, the acceptability rating of promising a toy as one of the reinforcement techniques decreased significantly after the course (P=0.01). Conversely, the acceptability of modeling as one of the desensitization techniques increased significantly, shifting from neutral to acceptable (P<0.0001).

Perceptions of pharmacological techniques were generally neutral before the course and showed a significant change in acceptance rates at the end of the course (P<0.0001). Acceptance of sedation and general anesthesia shifted significantly from neutral towards acceptable as shown in Table 2 (P<0.0001 and P=0.001, respectively). The use of nitrous oxide remained acceptable after the course and showed a significant change (P=0.01).

As shown in Table 2, before the course, the least acceptable techniques were aversive and communicative. Both showed significant change in their overall acceptance ratings after the course (P=0.04 and P<0.0001, respectively). However, with the exception of voice control, aversive techniques remained relatively unacceptable after the course though significant changes in perceptions were observed towards neutral (Table 2). Immobilization by staff or a parent decreased significantly in acceptability after the course (P=0.003). With the exceptions of stopping the child from speaking during treatment and mentioning the possibility of pain, all communicative behavior guidance techniques showed a significant increase in their acceptability, although they remained in the neutral range (P<0.0001). The acceptability of treating the child without local anesthesia increased significantly (P<0.0001), though it remained relatively unacceptable after the course.

Changes in acceptability towards other categories were also observed. Acceptability ratings of parent separation slightly increased. The presence of parents during their child’s treatment increased significantly in acceptability (P<0.0001). The acceptability of showing the needle to the child also increased significantly (P<0.0001), but remained strongly unacceptable after the course.

Analysis of the possible influence of demographic factors on the students’ perception scores indicated some statistically significant differences. Gender was found to have an influence on the students’ perceptions of several behavior guidance techniques. Table 3 shows the means and standard deviations of acceptability scores of different behavior guidance techniques and clinical situations for male and female students before and after the course. The mean changes in the acceptability scores of male and female students are also shown in Table 3. Before the course, female students rated some techniques as more acceptable than did male students, with significant differences between their scores as follows: modeling (P<0.0001), mentioning the possibility of pain (P=0.007), treatment without local anesthetic (P<0.0001), allowing the child to stop treatment (P<0.0001), having the parent present during treatment (P=0.001), and showing the needle to the child (P=0.003). However, after the course, several techniques were rated as more acceptable by male students than female students, with significant differences between their scores such as immobilization using a papoose board (P=0.013), treatment without local anesthetic (P=0.029), talking with the parent during treatment (P<0.0001), the parent talking with their child during treatment (P<0.0001), and having the parent present during treatment (P=0.013). Evaluating the changes in acceptability ratings across both genders revealed that several techniques showed a significant increase in their acceptability among male students when compared with the changes in their acceptability among female students. These techniques included immobilization using a papoose board (P=0.031), modeling (P<0.0001), mentioning the possibility of pain (P=0.013), treatment without local anesthetic (P<0.0001), allowing the child to stop treatment (P=0.002), talking with the parent during treatment (P=0.026), the parent talking with the child during treatment (P<0.001), having the parent present during treatment (P<0.0001), and showing the needle to the child (P=0.011).

**Table 3:** Means and standard deviations of pre- and post-course acceptability scores of male and female students and the mean changes in their perceptions

Technique or situation	Pre-course scores		Post-course scores		Change	
	Females	Males	Females	Males	Females	Males
	Mean ± SD	Mean ± SD	Mean ± SD	Mean ± SD	Mean ± SD	Mean ± SD
Reinforcement techniques						
Positive verbal reinforcement	4.34 ± 0.68	3.95 ± 0.95	4.23 ± 0.54	4.12 ± 0.90	−0.11 ± 0.83	0.16 ± 1.13
Promising a toy	4.03 ± 0.74	4.09 ± 0.75	3.51 ± 1.09	3.91 ± 0.78	−0.51 ± 1.19	−0.19 ± 1.02
Using the word “coward”	3.69 ± 1.02	3.40 ± 1.15	3.63 ± 1.06	3.58 ± 1.05	−0.06 ± 1.02	0.19 ± 1.11
Aversive techniques						
Voice control	3.46 ± 1.12	3.05 ± 1.21	3.77 ± 0.80	3.58 ± 0.98	0.31 ± 1.23	0.53 ± 1.05
Hand-over-mouth	1.69 ± 0.99	2.19 ± 1.13	2.09 ± 1.06	2.56 ± 1.24	0.40 ± 1.24	0.37 ± 1.60
Immobilization by staff or parent	2.57 ± 0.85	2.98 ± 0.88	2.26 ± 0.85	2.63 ± 0.90	−0.31 ± 0.96	−0.35 ± 1.11
Immobilization using papoose board	2.14 ± 0.87	2.16 ± 0.78	2.00 ± 0.80^b^	2.53 ± 1.00^b^	−0.14 ± 0.81^c^	0.37 ± 1.17^c^
Desensitization techniques						
Tell-show-do	4.17 ± 0.82	3.98 ± 1.03	4.06 ± 0.96	3.95 ± 1.13	−0.11 ± 1.07	−0.02 ± 1.53
Providing exact explanation	3.69 ± 0.71	3.74 ± 0.95	3.54 ± 0.91	3.77 ± 1.04	−0.14 ± 1.08	0.02 ± 1.22
Using music or video distraction	4.09 ± 0.78	3.93 ± 0.75	4.23 ± 0.77	4.02 ± 1.07	0.14 ± 0.73	0.07 ± 1.07
Using the child’s imagination	4.46 ± 0.65	3.95 ± 0.89	4.51 ± 0.50	3.84 ± 0.94	0.06 ± 0.68	−0.12 ± 0.95
Use of euphemisms	3.86 ± 0.94	3.93 ± 0.85	4.03 ± 0.78	3.93 ± 1.03	0.17 ± 0.82	0.00 ± 1.06
Modeling	3.23 ± 1.23^a^	2.28 ± 0.45^a^	3.80 ± 1.07	4.00 ± 0.84	0.57 ± 1.17^c^	1.72 ± 0.95^c^
Pharmacological techniques						
Nitrous oxide	3.60 ± 0.69	3.49 ± 0.82	3.91 ± 0.65	3.77 ± 0.94	0.31 ± 0.86	0.28 ± 1.07
Using sedation	2.74 ± 1.01	3.07 ± 0.93	3.34 ± 0.90	3.49 ± 0.96	0.60 ± 1.28	0.42 ± 1.07
General anesthesia	2.66 ± 0.87	2.79 ± 1.28	3.20 ± 1.02	3.05 ± 1.30	0.54 ± 1.35	0.26 ± 1.25
Communicative techniques						
Disallowing child speaking during treatment	2.43 ± 0.81	2.30 ± 0.88	2.26 ± 0.74	2.56 ± 1.05	−0.17 ± 1.04	0.26 ± 1.36
Mentioning the possibility of pain	3.46 ± 0.78^a^	3.07 ± 1.03^a^	3.29 ± 0.89	3.56 ± 0.73	−0.17 ± 1.20^c^	0.49 ± 1.09^c^
Treatment without local anesthetic	1.57 ± 0.69^a^	1.00 ± 0.00^a^	1.80 ± 0.86^b^	2.30 ± 1.08^b^	0.23 ± 0.91^c^	1.30 ± 1.08^c^
Allowing child to stop treatment	3.40 ± 0.97^a^	2.30 ± 0.59^a^	3.46 ± 1.17	3.23 ± 1.04	0.06 ± 1.30^c^	0.93 ± 1.14^c^
Dentist talks with parent during treatment	2.09 ± 1.09	2.35 ± 0.78	2.43 ± 0.91^b^	3.33 ± 0.99^b^	0.34 ± 1.16^c^	0.98 ± 1.28^c^
Dentist remains quiet during treatment	2.31 ± 1.10	2.07 ± 0.66	2.77 ± 0.91	2.72 ± 0.95	0.46 ± 1.44	0.65 ± 1.13
Parent talks with child during treatment	2.00 ± 0.90	2.21 ± 0.55	2.31 ± 0.83^b^	3.53 ± 0.73^b^	0.31 ± 1.13^c^	1.33 ± 1.01^c^
Clinical situations						
Parent separation	3.20 ± 1.10	2.72 ± 1.16	3.63 ± 0.94	2.79 ± 1.18	0.43 ± 1.22	0.07 ± 1.33
Parent present during treatment	3.03 ± 1.07^a^	2.40 ± 0.49^a^	3.31 ± 0.76^b^	3.48 ± 0.97^b^	0.29 ± 0.95^c^	1.44 ± 1.05^c^
Showing the needle to the child	1.23 ± 0.49^a^	1.00 ± 0.00^a^	1.34 ± 0.68	1.67 ± 1.06	0.11 ± 0.75^c^	0.67 ± 1.06^c^

*SD* standard deviation

Same letters in the horizontal rows indicate significant differences from each other. P<0.05 (analysis of variance)

Investigating the effect of other demographic factors on the students’ changes in acceptability ratings indicated that having a younger sibling was associated with a significant decrease in the acceptability of the tell-show-do technique (P=0.002). The acceptance of using sedation was significantly increased in married students with children (P=0.023). Mentioning the possibility of pain showed a significant increase in acceptability in students who had undergone unpleasant dental experiences (P=0.016). No other statistically significant correlations were observed between changes in acceptability ratings and having older siblings, having a dentist in the family, having previous dental or medical treatment, or having medical experience.

## Discussion

In the present study, students’ perceptions of behavior guidance techniques were obtained before and after a complete set of didactic lectures in behavior guidance techniques and after 1 academic year of clinical training in pediatric dentistry.

In the current study, timing was a critical issue that might have affected the students’ response rate. Response rate for the pre-course questionnaires was 84.5 %. Questionnaires were distributed on only one occasion before the course because it was crucial that responses were collected before the delivery of any lecture about behavior guidance techniques, and before the start of any clinical contact with the pediatric patients. The post-course questionnaires were distributed at the end of the academic year to ensure that all students had completed the didactic and clinical components of the pediatric dentistry course. At the end of the study, the students’ response rate had dropped to 67.25 %. This drop might be attributable to loss of interest among the students, over the course of the year they were asked to participate voluntarily in other studies and obliged to participate in the college’s surveys.

Students’ acceptability ratings of several techniques changed between the first and second time they filled out the questionnaire. In general, reinforcement and desensitizing behavior guidance techniques such as positive verbal reinforcement, promising a toy, tell-show-do, using the child’s imagination, and distraction were rated as most acceptable by Saudi fourth-year dental students. Similar findings have been reported by previous studies evaluating the perceptions of first-year dental students towards behavior guidance techniques [6, 7]. In another study, third-year dental students used reinforcement and desensitizing techniques in managing pediatric patients more than any other technique being taught in the dental school [12]. Additionally, most pediatric dentists and general practitioners surveyed in Saudi Arabia and other countries preferred and used these behavior guidance techniques [11, 13]. From the parents’ perspective, reinforcement and desensitizing behavior techniques were also ranked as highly acceptable techniques [10, 14]. After evaluating students’ perceptions for the second time, these techniques were still accepted by the students. However, a significant negative change was observed in students’ attitudes towards promising a toy and a significant positive change was observed in the acceptability of modeling. This might be related to the degree of effectiveness of these techniques in controlling or modifying child behavior when employed by the students in their practicing in clinics, which resulted in decreasing or increasing of their acceptability scores.

Pharmacological behavior guidance techniques were initially accepted by Saudi dental students to a lesser extent. This may be owing to a perception that psychological interventions have fewer possible side effects. Nitrous oxide was accepted more than conscious sedation or general anesthesia, which might have been influenced by their earlier introduction to nitrous oxide in other courses. This result is also in accordance with previous studies [6, 12]. However, significant positive changes were noticed in students’ perceptions of all pharmacological techniques, which might be owing to the effect of the didactic component of the course: they are not allowed to practice nitrous oxide, sedation, or general anesthesia at this level.

The least accepted behavior guidance techniques among Saudi dental students were aversive techniques, excluding voice control. Before the course, immobilization with staff or parents was viewed as more acceptable than immobilization using a papoose board; however, after the course, immobilization using a papoose board showed an increase in acceptability. Similar results were reported by Sotto et al. [6]. Although it showed a significant increase in acceptability, the hand-over-mouth technique was not initially accepted by the students, even before they were made aware that this technique was removed from the behavior guidelines of the AAPD. Earlier studies reported comparable results regarding the use of aversive techniques [6, 7]. However, a significant positive change was observed in students’ perceptions of voice control, which might be because of the perceived efficacy of this technique by students in controlling patients’ behavior.

Some communicative techniques and clinical situations were partially accepted and some were not accepted. The use of these techniques reflects the personality and communication skills of the users, who will need much more exposure to different clinical situations. This is corroborated by the fact that acceptance of these techniques was still in the neutral range after the educational course, even though significant changes were noted in the students’ perceptions of the communicative techniques. Treatment without a local anesthetic when it is refused by a child was not viewed as acceptable by the students before or after the course, presumably because students value local anesthesia in reducing pain and achieving better patient cooperation.

Students were initially neutral with regard to acceptance ratings of the presence of parents during dental treatment. However, their perceptions shifted significantly towards acceptance at the end of the course. This could be attributable to their appreciation of the importance of parents’ presence in maintaining, modifying, and controlling their children’s behavior that was observed during the clinical training. Most of the surveyed parents also preferred to stay with their children during the dental treatment [9, 10, 14]. Showing the needle to the child was also not acceptable, this approach might scare the children and lead to the loss of their cooperation. After the course, even though there was a significant positive change towards showing the needle to the child, this technique was still rated as unacceptable. This might be explained by the introductory clinic in which the students were taught to make every effort not to show the needle to the children to gain their cooperation. Previous studies have also reported a lack of acceptance of this technique by dental students [6, 7].

Regarding the differences between male and female students on the acceptance score changes, immobilization using a papoose board and modeling were rated as more acceptable by male students. This is in agreement with Sotto et al. [6] who found an increase in the acceptability of immobilization using a papoose board among male students. In a previous study investigating the difference in the use of behavior techniques by male and female dentists, it was reported that a papoose board was used more by male dentists than female dentists, whereas modeling was more widely used by female dentists [15]. Male students showed higher increases in acceptance ratings of more communicative behavior techniques than female students, which might be related to better communicative skills among male students. Male students showed more of an increase in acceptability ratings for the presence of the child’s parent during treatment than female students, which might be related to a difference in tolerance levels between male and female students of parental interference during dental treatment.

Concerning the effects of other demographic factors on students’ perceptions, the acceptability of using sedation was significantly increased in married students with children. This could be attributable to the fact that sedation was presented as a safe and efficient solution in managing pediatric dental patients. Students who had undergone unpleasant dental procedures themselves showed a significant increase in acceptability ratings of mentioning the possibility of pain. This might be related to the perception that patients should be made aware of the pain before they feel it. No other correlations were found between acceptability rating changes and other demographic factors. The small sample size may limit the interpretability of the analysis of variance findings with regard to the students’ demographic differences.

Although our results are consistent with previous findings, some factors should be considered when interpreting the results. These include the type and length of the curriculum, students’ academic level, educational materials, clinical training, qualifications, experience with the faculties, and the types of patients and parents. As this study was performed on a limited number of students studying the same curriculum, further studies should be conducted to assess the perception of other dental students who have different curricula in other dental colleges over the country.

## Conclusions

Generally, Saudi dental students rated most basic behavior management techniques such as reinforcement, desensitization, and communicative techniques as acceptable. Advanced behavior guidance techniques such as aversive and pharmacological techniques were viewed as less acceptable. An educational course, including didactic and clinical components, improved their level of acceptance and had a considerable influence on their perceptions of behavior guidance techniques in pediatric dentistry.

## Abbreviation

AAPD: American Academy of Pediatric Dentistry
